# Bone and Joint Tuberculosis in Greece: A Retrospective Study From 2011 to 2019

**DOI:** 10.7759/cureus.27033

**Published:** 2022-07-19

**Authors:** Irena Karabella, Efstathios Chronopoulos, George Panagiotakopoulos, Ismene A Dontas

**Affiliations:** 1 Infectious Diseases, Sotiria Thoracic Diseases Hospital of Athens, Athens, GRC; 2 Laboratory for Research of the Musculoskeletal System, KAT General Hospital, School of Medicine, National and Kapodistrian University of Athens, Athens, GRC; 3 Clinical Pharmacology, University of Patras, Patra, GRC

**Keywords:** greece, tuberculosis, lungs, joint, bone

## Abstract

Purpose: Bone and joint tuberculosis (BJTB) represents approximately 2-5% of all tuberculosis (TB) cases and its characteristics are infrequently discussed. The aim of this study was to examine the epidemiology, characteristics, and management of BJTB in Greece.

Methods: We conducted retrospective analyses of demographic clinical and treatment data accessed from medical records of patients with BJTB and with pulmonary involvement between 2011 and 2019 from the national TB reference hospital in Greece. Factors associated with treatment outcomes among patients were evaluated.

Results: Out of the total 3064 confirmed TB cases during the study period, 54 patients had either BJTB or skeletal TB with pulmonary involvement. The majority of patients were males (81.5%) and the mean age of the patients was 37.81±18.92 years. Out of the total patients, 11 were Greek and 43 were immigrants. Women had a higher probability of experiencing a stable or negative treatment outcome. Also, the patients that received therapy for less than nine months had 16.3 times higher probability of experiencing a stable or negative treatment outcome, compared to those that received therapy for more than 12 months.

Conclusions: The study indicated that the sex and the duration of treatment correlated with the treatment outcomes. It is essential to monitor and accurately report treatment outcomes so as to achieve disease control.

## Introduction

Tuberculosis (TB) is one of the oldest recognized diseases of mankind but is still a major public health issue in most of the world [1,2]. TB affects primarily the lungs and gastrointestinal tract, but may also infect the musculoskeletal system.

Bone and joint tuberculosis (BJTB) generally arises from reactivation of *Mycobacterium tuberculosis* that has migrated via blood circulation to the bone/joint tissues and remained there in latency, since the primary infection [3]. The disease represents a rare - yet clinically serious - extrapulmonary manifestation of the disease including pain, tenderness, and various limitations of motion, as well as other specific and general symptoms depending on the location, the staging, and the gravity of the disease [4,5]. The most commonly affected area is the spine, followed by some extremely susceptible extraspinal sites that include the synovial joints and the tendons [4,6,7]. Apart from lung, bone, and joint lesions, the disease might affect other areas as well, such as the skin, the abdomen, the lymph nodes, the digestive system, and the genitourinary tract [7-9].

Risk factors of TB include immunosuppressive conditions/drugs (such as HIV), comorbidities (diabetes, chronic renal/hepatic failure, chronic obstructive disease, lymphoproliferative disorders), young age as well as socioeconomic and behavioral factors, such as tobacco smoke, alcohol/drug use and poor living condition (e.g., overcrowded households, homelessness, malnutrition, low accessibility to healthcare services, poor personal hygiene) [4,10].

Effective antibiotic treatment is available, whereas consists of long and complex regimens. For patients with isoniazid- and rifampin-susceptible TB the recommended treatment duration is six to nine months while for patients with rifampin-monoresistant TB is less than 18 months, following culture conversion, and for patients with isoniazid- and rifampin-resistant TB is 18-24 months. In general, the preferred treatment duration for musculoskeletal tuberculosis is nine months [11].

BJTB has seen a significant increase during the latest years mostly due to the global increase of HIV infection, specifically in populations living in poor socio-economical conditions. The recently increased migration flows have also been a significant reason why the disease rates have risen alarmingly in developed countries. BJTB constitutes approximately 2-5% of all TB cases and 10% of extra-pulmonary TB cases, while at the same time, it represents 2.2-4.7% of all the tuberculosis cases in Europe and the USA and around 10-15% of extrapulmonary TB [3]. The incidence of BJTB has increased in the past two decades, especially in underdeveloped countries. In developed countries, 58-81% of skeletal TB cases are diagnosed in immigrants. Data from developing countries, such as Asia, indicate an incidence of extrapulmonary cases rising up to 15-20% [3,12]. As for the age distribution, most studies seem to agree that the disease follows a bimodal pattern (especially in developing countries), having the first peak during the ages 20-35 years, and the second peak during the ages older than 55 years [13-15]. Another important epidemiological factor that needs to be taken into account is the annual risk of infection from the Mycobacterium tuberculosis, which is under 0.5% in developed countries, but in Latin America and the Middle East, it rises to 1.5%, while in sub-Saharan countries of Africa it rises to 2.5%, which shows an indirect link among the socio-economic status and the prevalence of the disease [16].

The available epidemiological data for Greece is limited mainly due to the fact that the majority of the TB cases refuse to report it because of the fear of stigmatization, especially in the immigrant populations, that are most of the time already experiencing social exclusion and inequalities in most domains of their lives [17,18]. This lack of epidemiological data results in the absence of clear evidence about the duration of the therapy received, as well as about the percentages of the patients that experienced microbial resistance. Over the past decade, with the financial remission that has dominated in Greece and the significant increase of the emigrational flows, there are concerns about the numbers of tuberculosis incidents and thus, BJTB incidents as well.

This study aimed at shedding light on this significant research gap with the collection and statistical analysis of the data referring to the period 2011-2019.

## Materials and methods

This is a retrospective study of BJTB cases diagnosed in the Department of Thoracic Diseases General Hospital Sotiria from January 2011 to December 2019. Sotiria General Hospital is the national reference center for tuberculosis and mycobacteria and the leading hospital specializing in treating tuberculosis in Greece. In this study, all adult patients (inpatients and out­patients) who were diagnosed with bone, joint, and bone-lung TB during the study period were included.

The study was conducted in accordance with the World Medical Association Declaration of Helsinki-ethical principles for medical research involving human subjects. This study did not use identifiable private information or identifiable biospecimens. Approval for this study was obtained from the Ethics Committee of the “Sotiria” General Hospital (#139, 20/3/2019) and the National and Kapodistrian University of Athens (#109, 15/4/2019).

The following data were retrieved from the medical records of the patients: sex, age, site(s) of disease (bone/joint and concomitant pulmonary involvement), ethnic origin, comorbidities, TB treatment regimen, duration of treatment, multidrug-resistant (MDR)/extensively drug-resistant (XDR) - TB test results, neurological symptoms, surgical intervention, compliance to treatment, outcome of treatment and the diagnostic method (specimens/sputum collection). The diagnosis in the majority of cases was based mainly on positive cultivation of *M. tuberculosis* for TB but in its absence, other clinical characteristics were used to confirm the clinical suspicion.

Statistical analysis

Data were expressed as mean±standard deviation (S.D.) or median (in case of violation of normality) for continuous variables and as percentages for categorical data. The Kolmogorov-Smirnov test was utilized for normality analysis of the parameters.

Unifactorial analyses were made by using the Student's t-test or Mann-Whitney test in case of violation of normality and Fisher's exact test to analyze the relationship between the outcome variable (stable vs improvement status), and the quantitative and qualitative demographic and clinical variables, respectively.

Demographic and clinical variables were assessed in multifactorial binary logistic regression model with enter method (all variables are entered at the same time in the model) to identify independent demographic and clinical predictors of the outcome variable (stable vs improvement status). ORs and 95% CIs were reported for all variables in the multifactorial model.

All tests are two-sided; statistical significance was set at p< 0.05. All analyses were carried out using the statistical package SPSS v 21.00 (Somers, NY: IBM Corporation).

## Results

A total of 3064 TB cases from January 2011 to June 2019 were recorded. Among them, 31 patients had skeletal TB (bone and/or joint) and 23 had skeletal TB accompanied by TB in the lung (pulmonary involvement). Nine patients who met our inclusion criteria were excluded from further analysis because of missing data from their medical records. Thus, our final dataset for analysis was 54 cases.

The patients’ demographic data are presented in Table 1. The majority (81.5%) of patients were men while 18.5% were women. The mean age of the participants was 37.81±18.92 years. Out of the total 54 patients, 11 were of Greek origin and 43 were from foreign countries. For the purposes of analysis, Greek patients have been grouped together with the other Europeans, thus 33.3% were European and an equal percentage were of African and of Asian origin. As for the localization of the disease, 9.3% suffered from lesions on the cervical spine, 46.3% on the thoracic spine, 53.7% on the lumbar spine, and 29.6% of the lesions were not on the spinal column. As far as the Mycobacterium cultivation is concerned, 83.3% had a result of cultivation included in the diagnostic criteria; in 16.7%, it was not carried out, while 29.6% had other clinical criteria as diagnostic tools. Finally, regarding the cultivation result, 11.1% had a negative result and 72.2% had a positive result.

**Table 1:** Patients’ demographic data.

Demographics		N	%
Sex	Men	44	81.5
	Women	10	18.5
Ethnic origin	Asian	18	33.3
	African	18	33.3
	European	18	33.3
Site of TB	Bone/joint	31	57.4
	Bones and lung	23	42.6
Bone TB location	Cervical spine	5	9.3
	Thoracic spine	25	46.3
	Lumbar spine	29	53.7
	Appendicular skeleton	16	29.6
Culture-based diagnosis of TB	No	9	16.7
	Yes	45	83.3
Diagnosis of TB based on clinical criteria	No	38	70.4
	Yes	16	29.6
Culture results	Negative	6	11.1
	Positive	39	72.2
	Not carried out	9	16.7
Age (years)	Mean ± SD (min-max)	37.81 ± 18.92 (18-85)	

A percentage of 33.3% of the patients had significant comorbidities, among which 3.7% suffered from diabetes mellitus, 9.3% had already suffered from tuberculosis at a younger age, 3.7% suffered from chronic renal disease, 5.6% were immunosuppressed, 1.9% were HIV positive, 5.6% were hepatitis B virus (HBV)-positive/HBV-infected and 3.7% were hepatitis C virus (HCV)-positive (Table 2).

**Table 2:** List of patient comorbidities. HBV: hepatitis B virus; HCV: hepatitis C virus

Demographics		N (total = 54)	%
Comorbidities	No	36	66.7
	Yes	18	33.3
Diabetes mellitus	No	52	96.3
	Yes	2	3.7
History of tuberculosis	No	49	90.7
	Yes	5	9.3
Chronic renal disease	No	52	96.3
	Yes	2	3.7
Immunosuppression	No	51	94.4
	Yes	3	5.6
HIV	No	53	98.1
	Yes	1	1.9
HBV	No	51	94.4
	Yes	3	5.6
HCV	No	52	96.3
	Yes	2	3.7

The majority of the patients (55.6%) received treatment consisting of a two-month phase of four-drug regimens containing isoniazid, ethambutol, pyrazinamide, and rifampicin followed by a continuation phase of seven months of a two-drug regimen of isoniazid and rifampicin. The remaining 44.4% of patients received different drug regimens depending on their history of prior treatment for TB, the antibiotic sensitivity of *M. tuberculosis*, allergic or adverse reactions to previous treatment of TB, and poor or lack of compliance with previous TB treatment.

Further, 29.6% of patients received treatment for up to nine months, 53.7% received it for nine to 12 months, and 16.7% received it for more than 12 months. The majority of patients (75.9%) had a negative multidrug-resistant (MDR) TB, while the results were unknown or not adequately documented in a percentage of 18.5% of patients. At the same time, 1.9% had a positive extensively drug-resistant (XDR) TB, 79.6% had a negative XDR, and 18.5% results were not documented.

Treatment outcome assessment revealed that 41 (75.9%) patients had clinical improvement, while the remaining 13 (24.1%) patients had a stable or negative outcome. Regarding the other clinical variables examined, 11.1% were managed surgically, 9.3% wore an orthopedic brace, 27.8% had neurological symptoms and 83.3% had good compliance with the drug therapy. Last, the mean duration of the treatment received was 10.8 months, with the lowest duration of one month, and the highest of 21 months (Table 3).

**Table 3:** Clinical data of patients. MDR: multidrug-resistant; XDR: extensively drug-resistant

Demographics		N	%
Drug therapy	4-drug/ 2-drug regimen	30	55.6
	Other	24	44.4
Treatment duration	Less than 9 months	16	29.6
	9-12 months	29	53.7
	Over 12 months	9	16.7
MDR	No	41	75.9
	Yes	3	5.6
	Unknown	10	18.5
XDR	No	43	79.6
	Yes	1	1.9
	Unknown	10	18.5
Treatment outcome	Improvement	41	75.9
	Stable or negative	13	24.1
Surgical management	No	48	89.9
	Yes	6	11.1
Orthopedic brace	No	49	90.7
	Yes	5	9.3
Neurological symptoms	No	39	72.2
	Yes	15	27.8
Treatment compliance	No	9	16.7
	Yes	45	83.3
Duration of treatment	Mean ± SD (min-max)	10.78 ± 4.18 (1-21)	

The results presented in Table 4 show that there is a statistically significant impact of the variables “sex” (4.5 {1.1-19.3} p=0.048) and “duration of treatment” (up to nine months vs more than 12 months, 13.3 {1.3-134.6} p=0.028, and nine to 12 months vs more than 12 months, 0.9 {0.1-10.1}, p=0.923) on the outcome of treatment, whereas the other variables were not correlated by any means to the outcome of treatment.

**Table 4:** Unifactorial analysis of the outcome of treatment.

Demographics		Improvement, N%	Stable or negative outcome of treatment, N%	OR (95% CI)	p-Value
Sex	Men	36, 87.8	8, 61.5	4.5 (1.1-19.3)	0.048
	Women	5, 12.2	5, 38.5		
Origin	Asia	16, 39.0	2, 15.4	Ref.	0.297
	Africa	12, 29.3	6, 46.2	4.0 (0.7-23.4)	0.124
	Europe	13, 31.7	5, 38.5	3.1 (0.5-18.5)	0.220
Localization of disease	Bone/joint	23, 56.1	8, 61.5	0.8 (0.2-2.9)	0.730
	Bone/joint and lungs	18, 43.9	5, 38.5		
Comorbidities	No	27, 65.9	9, 69.2	0.9 ( 0.2-3.3)	0.822
	Yes	14, 34.1	4, 30.8		
Drug therapy	4-drug/2-drug regimen	22, 53.7	8, 61.5	0.7 (0.2-2.6)	0.619
	Other	32, 46.3	5, 38.5		
Duration of treatment	Less than 9 months	6, 15.0	10, 71.4	13.3 (1.3-134.6)	0.028
	9-12 months	26, 65.0	3, 21.4	0.9 (0.1-10.1)	0.923
	Over 12 months	8, 20.0	1, 7.1	Ref.	0.002
Surgical management	No	35, 87.5	13, 92.9	0.54 (0.1-5.1)	0.588
	Yes	5, 12.5	1, 7.1		
Orthopedic brace	No	37, 92.5	12, 85.7	2.1 (0.3-13.8)	0.458
	Yes	3, 7.5	2, 14.3		
Neurological symptoms	No	30, 75.0	9, 64.3	1.7 (0.5-6.1)	0.444
	Yes	10, 25.0	5, 35.7		
Age	-	37.5 ± 17.5	38.7±23.3	1.0 (0.97-1.04)	0.835

According to the multiple logistic regression for the variable “outcome of treatment”, there is a statistically significant effect of the participants’ sex and duration of the treatment on the outcome. Based on our results, women had 4.6 times a higher probability to have a stable or negative outcome of treatment compared to men. Moreover, patients who received therapy for less than nine months had 16.3 times a higher probability of having a stable or negative outcome versus to those who received therapy for more than 12 months (Table 5).

**Table 5:** Multifactorial analysis of the outcome of treatment.

Demographics		OR	95% CI		p-Value
Sex	Men	1.00	Ref.		0.097
	Women	4.57	0.76	27.49	
Duration of treatment	Less than 9 months	16.31	1.41	189.18	0.026
	9-12 months	1.09	0.09	13.17	0.941
	Over 12 months	1.00	Ref.		0.002

## Discussion

The economic crisis has reversed years of global progress in tackling tuberculosis which is a growing problem, especially in developing countries. BJTB is considered a diagnostic and treatment challenge since it is often overlooked. This study concerns BJTB cases between the years 2011 and 2019, a period of the global financial crisis and when Greece experienced major waves of immigration.

A study by Jutte et al. in 2004 showed an increasing trend of BJTB in the Netherlands during the period 1993-2000, reporting that the disease represented 4.3% of all TB cases [15]. Also, their origin was associated with the disease. Whereas, our findings showed that origin was not associated with BJTB, although the majority of cases were from foreign countries. Another significant conclusion of the study of Jutte et al. concerning the localization of the disease is that only 15% of BJTB patients also had pulmonary lesions while our results showed that 42.6% of BJTB patients had also pulmonary involvement.

A retrospective study by Mwachaka et al. in 2011 regarding spinal tuberculosis epidemiology among human immunodeficiency virus-negative patients in a Kenyan tertiary hospital within a period of five years showed that 77.5% of the patients presented back pain, and 72.9% limb weakness, while 61.2% of the patients exhibited significant neurological symptoms [19]. Based on the findings of our study, 75.9% of the patients had clinical improvement and 25% of those had neurological symptoms. Also, 25.6% of the total patients required surgical management whereas in our study that percentage was only 11.1%. Another study by De la Garza Ramos et al. in 2017 investigated the incidence of spinal tuberculosis in the United States between the years 2002 and 2011 [20]. The study concluded that 61% of the patients were male - 11.6% suffered from diabetes mellitus, 8.1% presented paralysis, and the most common location (61.9% of the patients) was at the thoracolumbar spine. These results are in line with ours, specifically, 81.5% of the patients were men, 3.7% suffered from diabetes mellitus, and that the most common TB site was at the thoracic (46.3%) and the lumbar (53.7%) spine.

Another study by Held et al. in 2017 assessed the distribution of age and localization of the disease in patients with musculoskeletal tuberculosis, the number of tuberculosis/HIV coinfections, as well as the incidence of multidrug-resistant (MDR) tuberculosis [21]. The results of the study showed that among the participants whose mean age was 27 years, spinal lesions were evident in 78% of the patients examined, 23% were HIV-positive and 4% showed MDR tuberculosis. Similarly, in our study, the mean age was 37.8 years, spinal lesions were in 70.4% of the patients, HIV-positives represented 1.9% of the patients and 5.6% had an MDR TB.

Golsha et al. in 2018 reviewed 229 skeletal tuberculosis cases in Northeast Iran during the period 2005-2014 [22]. The results showed that 56.3% of the participants were men, the mean age of the patients was 44 years, that spinal tuberculosis represented 81.2% of the patients, among which 96.9% reported pain as their main symptom.

Based on our results, sex and duration of treatment were associated with the outcome of treatment. Specifically, women had 4.6 times a higher probability to have a stable or negative outcome of treatment compared to men. Moreover, patients who received therapy for less than nine months had 16.3 times a higher probability of having a stable or negative outcome versus those who received therapy for more than 12 months. Our study showed that 75.9% had a favorable outcome which is in line with another study from Greece where the 73.2% of patients showed a favorable outcome for the years 2012-2017 [23].

## Conclusions

In conclusion, our study describes the epidemiological and clinical features of BJTB cases in Greece over the previous decade and brings to light important messages. A noteworthy sex difference was found, where women had a higher probability of experiencing a stable or negative treatment outcome The data also showed satisfactory clinical outcomes for patients that received prolonged anti-TB therapy. Thus, the main focus should be to raise public awareness, especially among vulnerable groups, about BJTB and the benefits of appropriate treatment. Several interventions need to be implemented so as to manage and control TB successfully. Integration of tuberculosis efficacious management services while national surveillance should be encouraged. The strategy of TB management should consist of preventing transmission, early detection, and appropriate treatment.
